# Later-Onset Hypertension Is Associated With Higher Risk of Dementia in Mild Cognitive Impairment

**DOI:** 10.3389/fneur.2020.557977

**Published:** 2020-11-26

**Authors:** Hongyun Qin, Binggen Zhu, Chengping Hu, Xudong Zhao

**Affiliations:** Shanghai Pudong New Area Mental Health Center, Tongji University School of Medicine, Shanghai, China

**Keywords:** mild cognitive impairment, hypertension, dementia, blood pressure, community

## Abstract

To investigate the correlation between hypertension development and the progression of mild cognitive impairment (MCI) to dementia in middle-aged and elderly people. A population-based longitudinal cognition survey of people aged 55+ was conducted. The hypertension onset age was estimated by self-reported information and medical insurance card records. To study the effect of later-onset hypertension on dementia, the incidence of dementia was compared between the two groups. Of 277 hypertensive MCI participants without dementia, 56 (20.22%) progressed to dementia (MCIp) over the 6-year follow-up. The proportion of MCIp participants in the old-age-onset hypertension group (≥65 years) was higher than that in the middle-age-onset hypertension group (27.0 vs. 15.4%, respectively; *X*^2^ = 5.538, *P* = 0.019). In the old-age-onset hypertension group, the proportion of MCIp without diabetes mellitus was higher than those with diabetes mellitus (24.7 vs. 12.6%, respectively; *X*^2^ = 5.321, *P* = 0.021) and those with increased pulse pressure was higher than those without increased pulse pressure (33.3 vs. 15.4%, respectively; *X*^2^ = 3.902, *P* = 0.048). However, the cox proportional hazard showed that older age was the only risk factor for MCIp (HR = 0.618, *p* = 0.000). These results suggest that individuals with later-onset hypertension may have greater cognition decline, even with blood pressure maintained at 130/80 mmHg with antihypertensive management.

## Introduction

With increases in population aging, the number of individuals with dementia continues to rise, with ~47 million people worldwide in 2015, which is expected to triple by 2050 (1). The two most common types of dementia are Alzheimer's disease (AD) and vascular dementia (VaD), accounting for ~80% (1–3). Mild cognitive impairment (MCI) is an intermediate transition stage between normal aging and dementia that is characterized by mild impairments in cognitive function without a decline in daily living or social functioning, and previous studies have shown that all MCI eventually progressed to dementia (MCI progression, MCIp) after 9 years (4). A multicenter study performed in China from 2008 to 2009 showed that the prevalence rates of MCI and dementia (in those >65 years old) were as high as 20.8 and 5.14%, respectively (5).

Hypertension is another disease that compromises the activites of daily living in the elderly population (6, 7). In the last 20 years, several studies have found that hypertension is a risk factor not only for AD but also for MCI (2, 7–10). In a 2-year follow-up study of 385 MCI patients, Goldstein et al. (11) showed that cognitive function (such as attention, executive function, and naming) of subjects with hypertension, compared to subjects without hypertension, significantly declined, especially in those with systolic blood pressure (SBP) ≥ 140 mmHg, or diastolic blood pressure (DBP) ≥ 90 mmHg. In other words, hypertension is a risk factor for MCIp. To date, there have been no confirmed effects of nootropic drugs on dementia, whereas hypertension is a very important controllable vascular risk factor (2). Therefore, it is crucial to quickly identify and manage high-risk patients with hypertension, which will subsequently prevent dementia.

The incidence of dementia has been related to the age of onset of hypertension. Numerous studies have shown that hypertension in middle-aged individuals is associated with increased dementia 20 or 30 years later, but the relationship to later-onset hypertension (≥65 years) is uncertain (9, 12). For example, some studies have found that hypertension is independently associated with cognitive impairment in later life and that cognitive function declines with increasing SBP, especially SBP ≥ 180 mmHg (13–15). In Yuan's study, the relationship between the decline in cognition as SBP increased was shaped like a “hockey stick (i.e., a gradual decline followed by a dramatic decrease)” (16). It was also confirmed in a cross-sectional survey that not only the proportion of hypertension (76.5 vs. 59.3%) but also the SBP level was higher in the dementia group than in the MCI group (15). However, some previous studies showed that hypertension did not necessarily increase the risk of dementia (2, 17–22). A 10-year follow-up (averaging 2.8 years) study of 559 individuals who were over 90 years old conducted by Corrada et al. (17) found that the risk of dementia in hypertensive patients aged 80 and 90 years was lower than that in non-hypertensive patients. Nevertheless, a high SBP level was possibly associated with an increased risk of dementia for individuals <75 years old, and this was not the case for those >80 years old (19–22). Moreover, it was not the high SBP level but the increased pulse pressure (PP) that was negatively correlated with cognition (23).

Our previous epidemiological investigation found that MCI in individuals with new-onset hypertension (≥60 years old) had a higher rate of dementia (24). We proposed the hypothesis that there may be a particular age (between 55 and 75 years old) when hypertension is particularly harmful to cognitive function. Our objective was to evaluate the association between the later onset of hypertension and the risk of all-cause dementia in a population-based cohort of individuals.

## Materials and Methods

### Ethics Statement

The research project was examined and approved by the Ethics Committee of the Mental Health Center of Pudong New Area, Shanghai (2017005). The guardians of all the dementia individuals provided ethical consent for their participation.

### Subjects

The current research was carried out in Pudong district in Shanghai. We analyzed elderly individuals with MCI (age ≥ 55 years old) who had participated in a survey from June 2011 to June 2012 (No. PKJ2010-Y26) and were successfully contacted for a subsequent survey in 2017. All participants signed informed consent forms. Only those sufficiently educated with adequate audiovisual skills that were needed to complete the necessary examinations were included.

Based on the age of onset of hypertension, the participants were divided into the old-aged group (onset age ≥ 65 years) and the middle-aged group (onset age <65 years).

### MCI and Dementia Diagnostic Criteria

MCI was diagnosed using the revised Mayo Clinic criteria: (1) elderly individuals consciously exhibited memory loss, especially those with memory impairment for more than 3 months; (2) overall cognitive function was normal through the Mini-Mental State Examination (MMSE total score: illiterate subjects > 17, with primary school education > 20 points, and others > 24 points); (3) clinical dementia rating (CDR) score reached a level of 0.5; (4) Montreal Cognitive Assessment Scale (MoCA) scores were ≤ 26; (5) the patient had normal function in daily life; and (6) the patient did not meet the diagnostic criteria for dementia (25, 26).

The criteria for dementia were as follows. (1) MMSE test scores were ≤ 17 for illiterate subjects, ≤ 20 points for subjects with primary school education, and ≤ 24 points for subjects with middle school education or above (27); (2) the subjects met the above criterion without definite blindness or speech difficulties.

### Cognitive and Other Neuropsychological Assessments

Subjects received cognitive and other neuropsychological assessments using the following scales where possible: (1) Mini-Mental State Examination (MMSE) scale (27), (2) Montreal Cognitive Assessment (MoCA) (26), (3) Hamilton Depression Scale (HAMD-17) (28), (4) Hachinski ischemic scale (HIS) (29), (5) activities of daily life scale (ADL) (30), and (6) clinical dementia rating (CDR) scale (31).

### Diagnosis and Treatment of Hypertension

The diagnosis, duration, age of onset, stage, and treatment of hypertension were collected based on self-reports from the elderly individuals and examination of their medical insurance card records. The treatment of hypertension was as follows, 34.0% (*n* = 91) patients take calcium channel blocker, 11.6% (*n* = 31) patients take angiotensin receptor blockers, and 7.1% (*n* = 19) patients take other medicines, whereas 41% (*n* = 110) patients unregularly take medicines; 6.3% (*n* = 17) patients take no medicines.

### Additional Variables

Other aspects of the medical histories reported at the baseline visit and considered potential confounders included stroke, transient ischemic attack, diabetes mellitus (DM), heart disease, and depression. Heart disease included any of the following diseases or surgeries: coronary artery disease, myocardial infarction, atrial fibrillation or other arrhythmias, heart valve disease, congestive heart failure, coronary artery bypass, or pacemaker placement. The highest level of education attained, marital status, and occupation were also recorded.

### Statistical Approach

Data were analyzed using the Statistical Package for Social Sciences (version 19.0; SPSS, IBM, Chicago, IL, USA). Continuous variables were tested for normality by the one-sample Kolmogorov-Smirnov-test. Continuous variables are expressed as the mean ± SD or median (range) as per distribution type, and categorical data are expressed as frequency and percentages. Statistical analyses were performed using independent samples *t*-tests for normal data and Mann-Whitney U-tests for non-normal data. Categorical data were analyzed by the chi-square-test. Cox proportional hazard was conducted to identify the determinants of the MCIp outcome. A *P*-value of < 0.05 was considered statistically significant.

## Results

### Follow-Up Outcomes of Hypertensive MCI Individuals in the Community

There were 56 individuals (20.22%) who progressed to dementia (MCIp), while 221 (79.78%) remained stable (MCIs). In comparison with the MCIs individuals, the MCIp individuals demonstrated significantly worse performance on the CDR and ADL scales (*p* < 0.01). In addition, the MCIp individuals were more likely to be single (*p* = 0.022; Table 1), which could be both the reason and consequence of cognitive function deterioration. Next, all individuals were divided into two groups according to the onset time of hypertension: the proportion of MCIp participants in the old-age-onset hypertension group (≥65 years) was significantly higher than that in the middle-age-onset hypertension group (27.0 vs. 15.4%, respectively; *X*^2^ = 5.538, *p* = 0.019). However, this may have been because the old-age-onset group were older than the middle-age-onset group (77.75 ± 6.75 vs. 70.45 ± 6.41, respectively, *p* < 0.001). For those without DM, the old-age-onset hypertension group showed a higher proportion of MCIp outcomes than the middle-age-onset group (26.5 vs. 10.6%, respectively; *X*^2^ = 9.071, *P* = 0.003) (Table 2), and the frequency of MCIp in those with increased pulse pressure was higher than in those without increased pulse pressure (33.3 vs. 15.4%, respectively; *X*^2^ = 3.902, *P* = 0.048). Here, increased pulse pressure refers to the value of systolic pressure minus diastolic pressure being higher than 60 mmHg. This result implied that in those individuals with a later onset of hypertension, increased pulse pressure was associated with the progression of cognitive impairment.

**Table 1:** Follow-up outcomes of hypertensive MCI patients.

**Variable**		**MCIp**	**MCIs**	**χ^**2/**t****^**	***P***
Age		72.75 ± 7.678	67.67 ± 6.968	4.772^a^	<0.001
Gender	Male	14 (19.7)	57 (80.3)	0.015^b^	0.904
	Female	42 (20.4)	164 (79.6)		
Singleness	Yes	16 (32.0)	34 (68.0)	5.252^b^	0.022
	No	40 (17.6)	187 (82.4)		
Educational level	Illiterate	23 (28.7%)	57 (71.3%)	6.347^b^	0.274
	Primary school	13 (14.6%)	76 (85.4%)		
	Junior school	17 (18.5%)	75 (81.5%)		
	High school	3 (23.1%)	10 (76.9%)		
	University	0 (0.0%)	1 (100.0%)		
	Post-graduate	0 (0.0%)	2 (100.0%)		
Smoking history	Yes	3 (16.7%)	15 (83.3%)	0.150^b^	0.698
	No	53 (20.5%)	206 (79.5%)		
Drinking history	Yes	2 (12.5%)	14 (87.5%)	0.627^b^	0.428
	No	54 (20.7%)	207 (79.3%)		
HAMD-17 score		2.34 ± 3.42	1.67 ± 2.14	1.406^a^	0.165
CDR score		0.75 ± 0.51	0.54 ± 0.35	−4.666^a^	<0.001
ADL score		18.04 ± 9.05	14.42 ± 2.07	2.973^a^	0.004
HIS score		2.96 ± 1.76	2.59 ± 1.07	1.512^a^	0.135
DM	Yes	17 (58.8)	44 (41.2)	2.840^b^	0.092
	No	39 (18.1)	177 (81.9)		
BMI		24.5 ± 3.5	24.6 ± 3.4	0.352^a^	0.725
SBP (mmHg)		131.48 ± 12.04	130.41 ± 11.57	0.613^a^	0.540
DBP (mmHg)		80.09 ± 7.10	79.81 ± 8.69	0.222^a^	0.824
PP (mmHg)		51.39 ± 12.00	50.60 ± 13.55	0.399^a^	0.690
Increased PP	Yes	20 (19.0)	68 (81.0)	0.504^b^	0.478
	No	36 (22.7)	153 (77.3)		

*MCIp, Mild Cognitive Impairment progression; MCIs, Mild Cognitive Impairment remained stable; DM, Diabetes Mellitus; BMI, Body Mass Index; SBP, Systolic blood pressure; DBP, Diastolic blood pressure; PP, pulse pressure = Systolic minus Diastolic pressure, Increased PP: PP > 60 mmHg in 2017*.

a *t-test*.

b *χ^2^-test*.

**Table 2:** Follow-up outcomes of hypertensive MCI stratified by age of onset.

**Variable**		**<65**		**χ^**2**^/*t***	***P***	**≥65**		**χ^**2**^/*t***	***P***
		**MCIp**	**MCIs**			**MCIp**	**MCIs**		
HAMD-17 score		2.80 ± 4.59	1.69 ± 2.21	1.188^a^	0.245	2.00 ± 2.05	1.64 ± 2.03	0.863^a^	0.390
CDR score		0.66 ± 0.40	0.52 ± 0.34	2.608^a^	0.009	0.81 ± 0.58	0.56 ± 0.36	3.559^a^	<0.001
ADL score		17.36 ± 8.50	14.40 ± 1.93	1.733^a^	0.096	18.44 ± 9.46	14.44 ± 2.27	2.368^a^	0.024
HIS score		3.00 ± 1.91	2.65 ± 1.15	0.886^a^	0.383	2.88 ± 1.66	2.53 ± 0.96	1.110^a^	0.274
Gender	Male	5 (13.5)	32 (86.5)			9 (26.5)	25 (73.5)	1.879^b^	0.170
	Female	20 (16.0)	105 (84.0)			22 (27.2)	59 (72.8)	3.772^b^	0.052
Singleness	Yes	4 (20.0)	16 (80.0)			12 (40.0)	18 (60.0)	2.206^b^	0.137
	No	21 (14.8)	32 (85.2)			19 (22.4)	66 (77.6)	2.096^b^	0.148
DM	Yes	12 (22.0)	101 (78.0)			8 (38.1)	13 (61.9)	1.819^b^	0.177
	No	9 (10.6)	114 (89.4)			27 (26.5)	75 (73.5)	9.071^b^	0.003
BMI		25.6 ± 3.2	25.2 ± 3.6	0.501^a^	0.617	23.8 ± 3.5	23.8 ± 2.9	0.047^a^	0.963
SBP (mmHg)		131.92 ± 13.20	131.01 ± 12.43	0.332^a^	0.740	130.78 ± 11.22	129.55 ± 10.02	0.543^a^	0.590
DBP (mmHg)		80.40 ± 5.94	80.90 ± 9.34	0.257^a^	0.798	79.84 ± 7.88	78.12 ± 7.21	1.125^a^	0.263
PP (mmHg)		51.52 ± 11.02	50.12 ± 15.44	0.434^a^	0.665	51.29 ± 12.91	51.39 ± 9.76	0.040^a^	0.968
Increased PP	Yes	8 (15.4)	44 (84.6)			12 (33.3)	24 (66.7)	3.902^b^	0.048
	No	17 (15.5)	93 (84.5)			19 (24.1)	60 (75.9)	2.203^b^	0.138

*MCIp, Mild Cognitive Impairment progressed to dementia; MCIs, Mild Cognitive Impairment remained stable; DM, Diabetes Mellitus; BMI, Body Mass Index; SBP, Systolic blood pressure; DBP, Diastolic blood pressure; PP, pulse pressure = Systolic minus Diastolic pressure, Increased PP: PP > 60 mmHg in 2017*.

a *t-test*.

b *χ^2^-test*.

### Cox Proportional Hazard of Cognitive Function Deterioration in MCI

**Figure 1:**
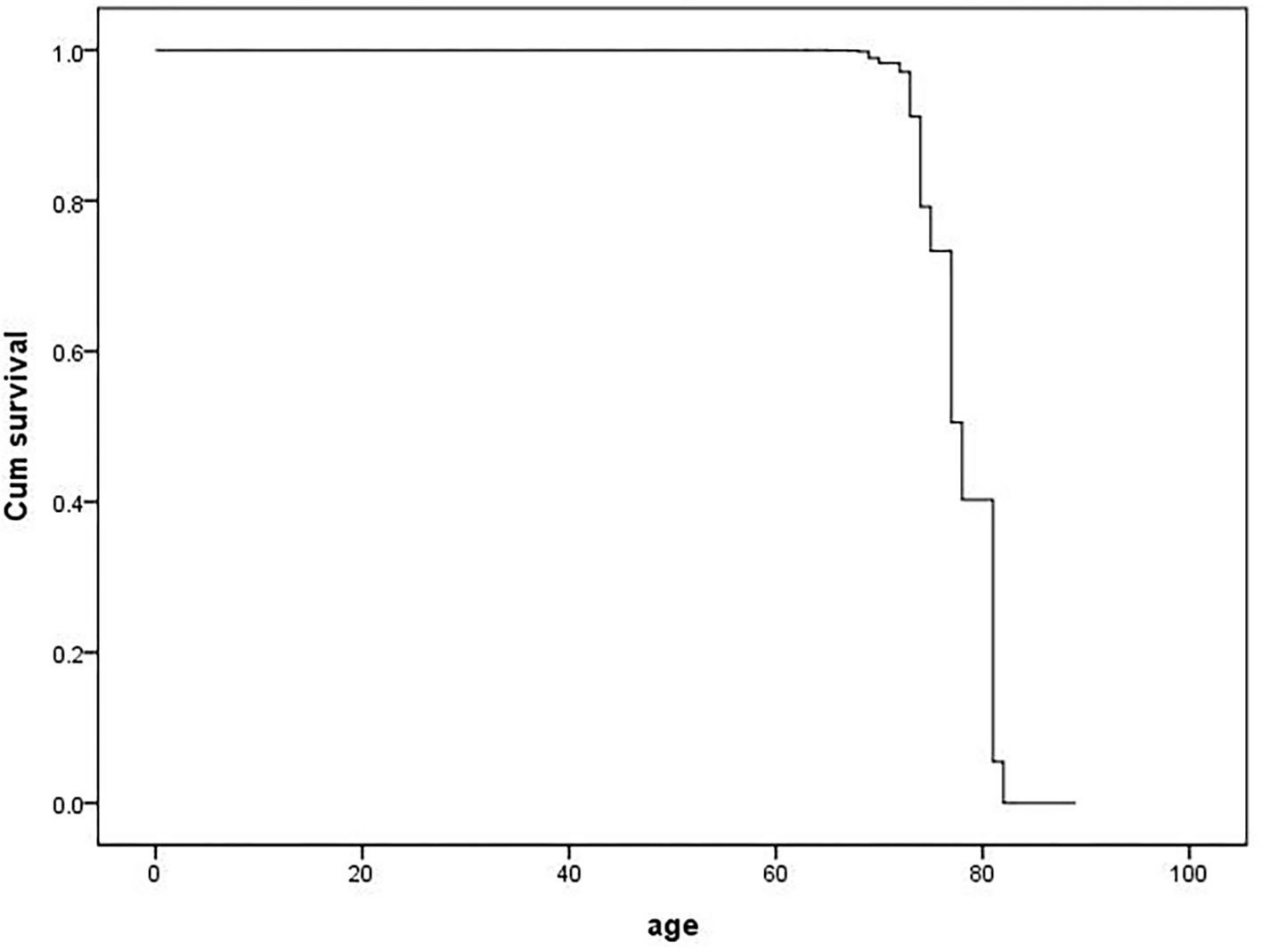
Survival function at mean of covariates.

The cox proportional hazard model was fitted to analyze the determinants of the MCI outcome at follow-up (MCIp = 0, MCIs = 1). Demographic data and variables such as hypertension, age at onset of hypertension, blood pressure grouping, and pulse pressure were taken as independent variables in the model. The variable assignment for the cox proportional hazard analysis is listed in Table 3. The results showed that age (*P* = 0.000, HR = 0.618) and BMI (*P* = 0.024, HR = 1.151) were the independent determinant of MCI outcome at follow-up and that patients with a age <80 and higher BMI were more likely to maintain cognitive function (Table 4 and Figure 1). Other factors demonstrated no significant association with MCIp (*P* > 0.05).

**Table 3:** The variables assignment of cox proportional hazard.

**Variable**	**Factors**	**Assignment**
X1	Gender	Male = 0, Female = 1
X2	Educational level	<12 years = 0, ≥12 years = 1
X3	Singleness	No = 0, Yes = 1
X4	Occupation	Mental labor = 1, Physical labor = 2
X5	Diabetes mellitus	No = 0, Yes = 1
X6	Hyperlipidemia	No = 0, Yes = 1
X7	Smoking	No = 0, Yes = 1
X8	Alcohol consumption	No = 0, Yes = 1
X9	History of dementia	No = 0, Yes = 1
X10	Hypertension alone	No = 0, Yes = 1
X12	Hypertension age of onset	≥65 years = 0, <65 years = 1

**Table 4:** Results of cox proportional hazard.

	**β**	**S.E**.	***P***	**HR**	**95% C.I. of HR**	
					**Lower limit**	**Upper limit**
Gender	−0.004	0.603	0.994	0.996	0.305	3.248
Education level	0.659	1.269	0.603	1.934	0.161	23.254
Age	−0.482	0.101	0.000^**^	0.618	0.506	0.754
Singleness	0.090	0.570	0.874	1.094	0.358	3.342
Occupation	0.192	0.655	0.770	1.211	0.335	4.375
Number of children	0.417	0.240	0.082	1.517	0.949	2.428
Diabetes mellitus	−0.864	0.792	0.275	0.421	0.089	1.991
Hyperlipidemia	−2.235	1.270	0.079	0.107	0.009	1.291
Smoking	0.914	1.009	0.365	2.493	0.345	18.029
Alcohol Consumption	−0.675	1.208	0.577	0.509	0.048	5.440
History of dementia	−0.406	0.730	0.578	0.667	0.159	2.785
BMI	0.141	0.062	0.024^*^	1.151	1.091	1.301
Hypertension alone	−1.193	0.845	0.158	0.303	0.058	1.590
Systolic pressure	−0.015	0.026	0.578	0.985	0.936	1.038
Pulse pressure	0.031	0.020	0.121	1.031	0.992	1.072
Hypertension age of onset	0.157	0.610	0.796	1.171	0.354	3.866

* *p < 0.05*,

** *p < 0.01*.

## Discussion

### Main Findings

#### The Proportion of MCIp Was Higher in the Group With Hypertension Onset ≥ 65 Years

In this prospective study of 277 MCI participants aged 73.48 ± 7.47 years having a late onset of hypertension (≥65 years) was related to a higher dementia risk compared with those with an onset of hypertension in middle age. This increased risk was limited to participants who reported a mean SBP of 130/80 mmHg. In addition, the differences disappeared after stratification according to age and BMI, SBP, and DBP levels. To our knowledge, our study is the first to report blood pressure levels and to include hypertension by age of onset.

Some researchers have found that an increased risk of dementia was related to the increase in SBP in the group under 75 years old (17). The risk of cognitive impairment increased by 1.17 times when SBP was 130–139 mmHg; however, the risk increased to 1.54 times when SBP was ≥180 mmHg (16). The results from a 3.8-year follow-up of individuals with hypertension (*n* = 2,800) showed that incidences of MCI and dementia decreased by 15% with intensive control of blood pressure (SBP < 120 mmHg) compared with the standard control group (SB < 140 mmHg) (HR = 0.85, 95% CI = 0.74–0.97, *P* = 0.02) (32); moreover, a statistically significant reduced risk of MCI and dementia was found in the intensive group (SBP = 120 mmHg) <75 years old (33).

The results of this study are not consistent with some previously published results. The reasons may be as follows: First, the average age of the participants in this study was 73 years with a mean SBP of 130/80 mmHg, 57% of them were <75 years old, 8 patients (2.89%) had SBP < 120 mmHg and 8 (2.89%) had SBP ≥ 160 mmHg. Our sample size was relatively small. Second, through medication management with a goal in the normal range (130/80 mmHg), the proportion of MCIp was different between the two groups, suggesting that aggressively lowering SBP may not be beneficial in elderly individuals (2). Similar findings were found in a long-term large-sample study (*n* = 1,440; 8-year follow-up) where the risk of dementia increased by 2.4 times when blood pressure was <140/90 mmHg in middle- to old-aged individuals with hypertension.

Furthermore, some studies have found that the relationship between the risk of dementia and the increase in SBP was not yet clear for those older than 85 years (17). After 10 years of follow-up (average 2.8 years, *n* = 559), Corrada et al. (17) reported that the risk of dementia in patients with new-onset hypertension and aged 80+ and 90+ years was lower than that in non-hypertensive patients (HR 0.54 and 0.37, *P* = 0.04 and 0.004, respectively). The results suggested that the relationship between hypertension and dementia in the elderly cohort was different from that in the middle-aged cohort. Hypertension may be a result of compensatory responses of the body. That is, the etiology of hypertension is similar to that of dementia, but the clinical symptoms occur at different times.

In addition, the cognitive impacts of middle-aged hypertension can only be followed up and analyzed after excluding cerebrovascular accidents (21); that is, there is bias in the samples. Patients with middle-aged-onset hypertension have severe conditions that make it difficult to detect the impact of hypertension on AD because of cerebrovascular damage such as stroke (22). In addition, other community-based studies have found that middle-aged hypertension was associated with MCI and dementia at the age of 70–90 (more strongly associated with dementia) (23). These data suggest that this part of the population did not go through the MCI stage and directly entered the dementia stage, which may have contributed to sample bias.

However, the results of the cox proportional hazard analysis failed to identify age of hypertension onset, blood pressure and increased pulse pressure as risk factors for MCI deterioration, and only older age was identified as a risk factor. This is consistent with the results of other studies confirming that old age is the main risk factor for dementia (34). We found that the proportion of MCIp was higher in the group with hypertension onset ≥ 65 years. It is worth mentioning that univariate analyses and cox proportional hazard analyses can have inconsistent conclusions. When comparing the two analysis methods, the results implied that there was indeed an association between onset age and MCIp, but the relationship was not strong enough to be observed in both methods. However, this is the first study reporting that the age of hypertension onset is a risk factor, and we believe that when more data are accumulated, this variable can be used to help predict MCIp.

#### The Proportion of MCIp Was Higher in the Group Without Diabetes Mellitus

Another finding of our study was that for hypertensive MCI individuals without DM, the incidence of MCIp in the old-age group was 1.96 times higher than that in the middle-age group. One meta-analysis also found that the ability of cerebrovascular risk factors to predict dementia/AD in the old-age group (average age of 72.3–82.5 years) was significantly reduced compared with younger age groups (35). That is, cerebrovascular diseases such as hypertension, diabetes, and hyperlipidemia did not necessarily increase the risk of dementia. A large-sample survey of community-based individuals in a brain magnetic resonance imaging (MRI) follow-up study (*n* = 2,367) revealed that cerebrovascular risk factors such as white matter degeneration, hypertension, diabetes, and smoking were associated with aging and contributed to dementia, although the age range of this survey was 20–90 years (19). It should be noted that only 61 cases (22.02%) of DM in this study may have resulted in an insufficient sample size, which made it difficult to identify significant differences. In addition, in individuals with MCI aged 73.48 (SD = 7.47) years, the pathogenesis of DM may be different from that in middle-aged individuals, so the effect of DM on dementia may also be different.

#### The Association Between MCIp and Higher Pulse Pressure

We noticed that the incidence of MCIp was higher in the groups of patients that had higher pulse pressure. Pulse pressure is a sign of arterial stiffness. It was previously shown that pulse pressure was associated not with hypertension and apolipoprotein E4 but with the deposition of beta-amyloid plaques in the brain (23). In other words, pulse pressure is closely related to aging.

Jefferson et al. (36) used pulse wave velocity (PWV, m/sec) to measure aortic stiffness and found that decreases in regional cerebral blood flow were related to increases in arterial stiffness despite the existence of cerebral blood flow reserve capacity. Follow-up and cross-sectional clinical studies have confirmed that increased pulse pressure increases the risk of dementia (including vascular dementia and AD), which indicates that medications may lead to occult hypotension and cerebral hypoperfusion (37).

#### MCIp Did Not Benefit From Antihypertensive Therapy

Regarding the prevention of dementia by antihypertensive therapy, some studies have suggested that the risk of dementia was increased by the potential hypotension induced by antihypertensive therapy due to impaired vascular regulation mechanism in elderly individuals (38–40). A 16-week follow-up study suggested that people over 75 years old with MCI who stopped taking antihypertensive drugs did not develop cognitive function deterioration (40). They did not explicitly state whether all MCI cases had late-onset hypertension, and their follow-up time was too short. A multicenter study found that hypertensive patients over 65 years of age showed a temporary increase in blood pressure 4 months after reducing antihypertensive medicine but recovered to 134 mmHg in 9 months, while the control group (without reducing drugs) had an increased risk of emergency hospitalization (20). Animal experiments have also suggested that sartan therapy can improve cognition in aged rats (41, 42). However, clinical studies, as well as meta-analyses, have reported that although antihypertensive therapy can reduce systolic or DBP, it does not reduce the incidence of dementia (12, 17, 23, 43). Taken together, for the prevention of dementia in elderly individuals with hypertension, there are many studies that still need to be conducted.

Besides, the result suggested that participants with higher BMI were more likely to maintain cognitive function. However, it is still early to tell the parameter can be protective factors, we cannot distinguish the actual BMI and the BMI decrease due to osteoporosis and malnutrition, which can confound the treatment of hypertension and DM.

## Conclusion

These results suggest that individuals with later-onset hypertension may have greater cognition decline, even in patients with blood pressure maintained at 130/80 mmHg with antihypertensive management.

### Research Limitations

First, 277 elderly people with hypertension in the MCI community were followed up prospectively. The sample size was small, and the age distribution was uneven. The follow-up time was 6 years. There might have been sample bias that affected the proportion of MCIp to some extent.

Second, the survey of occupational mental activity, post-retirement economic life, lifestyle, and other factors in the MCI population with hypertension was not detailed enough, and the influence of an insufficient sample size also had some influence on the results of the study.

Third, the diagnosis of dementia in this study sample did not combine detection with imaging and cerebral spinal fluid (CSF) biochemical indicators without classification by Alzheimer's disease, Lewy bodies dementia, Dementia associated with parkinsonism, etc. And the mental state assessed without a “comprehensive” neuropsychological battery. The history and examination of hypertension were mainly provided by the community elderly individuals and medical record cards. There was no 24-h ambulatory blood pressure monitoring, which may have introduced some bias and had a certain impact on the objectivity of the results. So there were several confounders not considered in the analysis (i.e., serum lipids concentration, smoking, and white matter lesions burden at the MRI).

### Research Significance

First, the results of the current investigation showed that MCI patients with hypertension with an average age of ~73 years old were prone to develop dementia, which suggests that the administration of antihypertensive drugs for hypertension in this population (≥65 years old) should be comprehensively considered. Because of the different mechanisms underlying hypertension, maintaining blood pressure at 130/80 mmHg may not have a positive effect regarding the protection of cognitive function. Second, this study showed that pulse pressure had a negative effect regarding the protection of cognitive function. In the group with increased pulse pressure, the proportion of the elderly patients progressing to dementia was higher than that of the middle-aged patients. This suggests that the pathogenesis of hypertension in older patients may be different than in middle-aged patients and that the effect of increased pulse pressure on cerebrovascular function also increased with age. Therefore, increased pulse pressure may be one of the risk factors for dementia. In the future, it is necessary to expand the research sample size and improve the experimental methods for further confirmation.

## Data Availability Statement

The raw data supporting the conclusions of this article will be made available by the authors, without undue reservation.

## Ethics Statement

The studies involving human participants were reviewed and approved by the Ethics Committee of the Mental Health Center of Pudong New Area, Shanghai (2017005). The patients/participants provided their written informed consent to participate in this study.

## Author Contributions

HQ and CH guided the implementation of the research, collated, and analyzed the data. XZ and BZ were the leaders. BZ gave essential points for the analysis of data. XZ was responsible for the quality control. All authors have read and approved the manuscript and ensure that this is the case.

## Conflict of Interest

The authors declare that the research was conducted in the absence of any commercial or financial relationships that could be construed as a potential conflict of interest.

## Abbreviations

AD: Alzheimer's disease
VaD: vascular dementia
MCI: mild cognitive impairment
MCIp: MCI progression
MCIs: MCI remaining stable
SBP: systolic blood pressure
DBP: diastolic blood pressure
PP: pulse pressure
MMSE: Mini-Mental State Examination
MoCA: Montreal Cognitive Assessment Scale
CDR: clinical dementia rating
HAMD-17: Hamilton Depression Scale
HIS: Hachinski ischemic scale
ADL: activities of daily life scale
DM: diabetes mellitus
BMI: body mass index
MRI: magnetic resonance imaging
PWV: pulse wave velocity
CSF: cerebral spinal fluid.

## References

[1] Livingston G Sommerlad A Orgeta V Costafreda SG Huntley J Ames D. Dementia prevention, intervention, and care. Lancet. (2017) 390:2673–734. 10.1016/S0140-6736(17)31363-6 28735855

[2] Tadic M Cuspidi C Hering D. Hypertension and cognitive dysfunction in elderly: blood pressure management for this global burden. BMC Cardiovasc Disord. (2016) 16:208. 10.1186/s12872-016-0386-0 27809779 PMC5093934

[3] Chan KY Wang W Wu JJ Liu L Theodoratou E Car J. Epidemiology of Alzheimer's disease and other forms of dementia in China, 1990–2010: a systematic review and analysis. Lancet. (2013) 381:2016–23. 10.1016/S0140-6736(13)60221-4 23746902

[4] Ward A Arrighi HM Michels S Cedarbaum JM. Mild cognitive impairment: disparity of incidence and prevalence estimates. Alzheimers Dement. (2012) 8:14–21. 10.1016/j.jalz.2011.01.002 22265588

[5] Jia J Wang F Wei C Zhou A Jia X Li F. The prevalence of dementia in urban and rural areas of China. Alzheimers Dement. (2014) 10:1–9. 10.1016/j.jalz.2013.01.012 23871765

[6] Sierra C Doménech M Camafort M Coca A. Hypertension and mild cognitive impairment. Curr Hypertens Rep. (2012) 14:548–55. 10.1007/s11906-012-0315-2 23073614

[7] Lazo-Porras M Ortiz-Soriano V Moscoso-Porras M Runzer-Colmenares FM Málaga G Jaime Miranda J. Cognitive impairment and hypertension in older adults living in extreme poverty: a cross-sectional study in Peru. BMC Geriatr. (2017) 17:250. 10.1186/s12877-017-0628-8 29073885 PMC5659043

[8] Zuo M Gan C Liu T Tang J Dai J Hu X. Physical predictors of cognitive function in individuals with hypertension: evidence from the CHARLS basline survey. West J Nurs Res. (2019) 41:592–614. 10.1177/0193945918770794 29742988

[9] Wei J Yin X Liu Q Tan L Jia C. Association between hypertension and cognitive function: a cross-sectional study in people over 45 years old in China. J Clin Hypertens (Greenwich). (2018) 20:1575–83. 10.1111/jch.13393 30259624 PMC8031190

[10] Smith EE Muzikansky A McCreary CR Batool S Viswanathan A Dickerson BC. Impaired memory is more closely associated with brain beta-amyloid than leukoaraiosis in hypertensive patients with cognitive symptoms. PLoS ONE. (2018) 13:e0191345. 10.1371/journal.pone.0191345 29381739 PMC5790236

[11] Goldstein FC Levey AI Steenland NK. High blood pressure and cognitive decline in mild cognitive impairment. J Am Geriatr Soc. (2013) 61:67–73. 10.1111/jgs.12067 23301925 PMC3699694

[12] Harrison JK Van Der Wardt V Conroy SP Stott DJ Dening T Gordon AL. New horizons: the management of hypertension in people with dementia. Age Ageing. (2016) 45:740–6. 10.1093/ageing/afw155 27836926 PMC5863782

[13] Gorelick PB Scuteri A Black SE Decarli C Greenberg SM Iadecola C. Vascular contributions to cognitive impairment and dementia: a statement for healthcare professionals from the american heart association/american stroke association. Stroke. (2011) 42:2672–713. 10.1161/STR.0b013e3182299496 21778438 PMC3778669

[14] Haring B Leng X Robinson J Johnson KC Jackson RD Beyth R. Cardiovascular disease and cognitive decline in postmenopausal women: results from the women's health initiative memory study. J Am Heart Assoc. (2013) 2:e000369. 10.1161/JAHA.113.000369 24351701 PMC3886762

[15] Liang X Shan Y Ding D Zhao Q Guo Q Zheng L. Hypertension and high blood pressure are associated with dementia among chinese dwelling elderly: the Shanghai aging study. Front Neurol. (2018) 9:664. 10.3389/fneur.2018.00664 30233479 PMC6131189

[16] Yuan JQ Lv YB Chen HS Gao X Yin ZX Wang WT. Association between late-life blood pressure and the incidence of cognitive impairment: a community-based prospective cohort study. J Am Med Dir Assoc. (2019) 20:177–82.e2. 10.1016/j.jamda.2018.05.029 30017702 PMC7150587

[17] Corrada MM Hayden KM Paganini-Hill A Bullain SS DeMoss J Aguirre C. Age of onset of hypertension and risk of dementia in the oldest-old: the 90+ study. Alzheimers Dement. (2017) 13:103–10. 10.1016/j.jalz.2016.09.007 28108119 PMC5318224

[18] Alzheimer's Association Alzheimer's disease facts and figures. Alzheimers Dement. (2017) 13:325–73. 10.1016/j.jalz.2017.02.001

[19] Peng SL Chen X Li Y Rodrigue KM Park DC Lu H. Age-related changes in cerebrovascular reactivity and their relationship to cognition: a four-year longitudinal study. Neuroimage. (2018) 174:257–62. 10.1016/j.neuroimage.2018.03.033 29567504 PMC5949266

[20] Gulla C Flo E Kjome RL Husebo BS. Deprescribing antihypertensive treatment in nursing home patients and the effect on blood pressure. J Geriatr Cardiol. (2018) 15:275–83. 10.11909/j.issn.1671-5411.2018.04.011 29915617 PMC5997621

[21] Sharifi F Hedayat M Fakhrzadeh H Jafar Mahmoudi M Ghaderpanahi M Mirarefin M Hypertension and cognitive impairment: Kahrizak elderly study. Int J Gerontol. (2011) 5:212–6. 10.1016/j.ijge.2011.12.001

[22] Alzheimer's Association Alzheimer's disease facts and figures. Alzheimers Dement. (2018) 14:367–429. 10.1016/j.jalz.2018.02.001

[23] Lima NKC. Hypertension and cognition decline: is there an ultimate link? J Clin Hypertens (Greenwich). (2018) 20:1584–6. 10.1111/jch.13401 30259645 PMC8031056

[24] Qin HY Zhu BG Wang L Guo Y Cao ZC Hu CP Follow-up investigation of the effect of the new-onset and untreated hypertension on the elderly patients with mild cognitive impairment in community. Chin Gen Pract. (2020) 23:1640–53. 10.12114/j.issn.1007-9572.2019.00.785

[25] Luis CA Loewenstein DA Acevedo A Barker WW Duara R. Mild cognitive impairment: directions for future research. Neurology. (2003) 61:438–44. 10.1212/01.WNL.0000080366.90234.7F 12939414

[26] Chen J Ye ZR Yuan M Fang Y The application and progression of the montreal cognitive assessment in mild cognitive impairment. Chin J Psvchiatr. (2017) 50:386–9. 10.3760/cma.j.issn.1006-7884.2017.05.01

[27] Gao MY Yang M Kuang WH Qiu PY Department of Epidemiology and Biostatistics West China School of Public Health Mental state examination in Chinese elderly people. J Peking Univ (Health Sci). (2015) 47:443–9. 10.3969/j.issn.1671-167X.2015.03.014 26080873

[28] Li F Bo QJ Zhao Y Liu H Wang CY The 3rd Ward of Beijing Anding Hospital Quality of life and related factors in patients with major depressive disorde. J Cap Med Univ. (2017) 38:186–91. 10.3969/j.issn.1006-7795.2017.02.008

[29] Zhang Q Dai CB Wang W Chen H Study on the relationship between dementia and plasma homocysteine level. Pract Geria. (2017) 29:134–6. 10.3969/j.issn.1003-9198.2015.02.015

[30] Yuan Quan LX Yao WB The demandations and factors for long-term care in institutions for disabled elderly. Chin J Gerontol. (2017) 6214–6. 10.3969/j.issn.1005-9202.2017.24.091

[31] Woolf C Slavin MJ Draper B Thomassen F Kochan NA Reppermund S. Can the clinical dementia rating scale identify mild cognitive impairment and predict cognitive and functional decline? Dement Geriatr Cogn Disord. (2016) 41:292–302. 10.1159/000447057 27332560

[32] Kjeldsen SE Narkiewicz K Burnier M Oparil S. Intensive blood pressure lowering prevents mild cognitive impairment and possible dementia and slows development of white matter lesions in brain: the SPRINT memory and cognition IN decreased hypertension (SPRINT MIND) study. Blood Press. (2018) 27:247–8. 10.1080/08037051.2018.1507621 30175661

[33] Zhu R Liu X He Z. Association between CLU gene rs11136000 polymorphism and Alzheimer's disease: an updated meta-analysis. Neurol Sci. (2018) 39:679–89. 10.1007/s10072-018-3259-8 29396813

[34] Liang X Chen Z Dong X Zhao Q Guo Q Zheng L. Mental work demands and late-life cognitive impairment: results from the Shanghai aging study. J Aging Health. (2019) 31:883–98. 10.1177/0898264318765034 29661060

[35] Hou XH Feng L Zhang C Cao XP Tan L Yu JT. Models for predicting risk of dementia: a systematic review. J Neurol Neurosurg Psychiatry. (2019) 90:373–9. 10.1136/jnnp-2018-318212 29954871

[36] Jefferson AL Cambronero FE Liu D Moore EE Neal JE. Higher aortic stiffness is related to lower cerebral blood flow and preserved cerebrovascular reactivity in older adults. Circulation. (2018) 138:1951–62. 10.1161/CIRCULATIONAHA.118.032410 30018169 PMC6394409

[37] Oishi E Ohara T Sakata S Fukuhara M Hata J Yoshida D Day-to-day blood pressure variability and risk of dementia in a general japanese elderly population: the Hisayama study. Circulation. (2017) 136:516–25. 10.1161/CIRCULATIONAHA.116.025667 28784822 PMC5548511

[38] Streit S Poortvliet RKE Gussekloo J. Lower blood pressure during antihypertensive treatment is associated with higher all-cause mortality and accelerated cognitive decline in the oldest-old-data from the Leiden 85-plus study. Age Ageing. (2018) 47:545–50. 10.1093/ageing/afy072 29741555

[39] Momtaz YA Hamid TA Haron SA Bagat MF Mohammadi F. Prevalence of hypotension and its association with cognitive function among older adults. Aging Ment Health. (2018) 22:447–52. 10.1080/13607863.2016.1268093 28060530

[40] van der Wardt V Burton JK Conroy S Welsh T Logan P Taggar J. Withdrawal of antihypertensive therapy in people with dementia: feasibility study. Pilot Feasibility Stud. (2018) 4:29. 10.1186/s40814-017-0221-0 29340166 PMC5759798

[41] Riley DE Espay AJ. Cognitive fluctuations in Parkinson's disease dementia: blood pressure lability as an underlying mechanism. J Clin Mov Disord. (2018) 5:1. 10.1186/s40734-018-0068-4 29456869 PMC5811960

[42] Petek B Villa-Lopez M Loera-Valencia R Gerenu G Winblad B Kramberger MG. Connecting the brain cholesterol and renin-angiotensin systems: potential role of statins and RAS-modifying medications in dementia. J Intern Med. (2018) 284:620–42. 10.1111/joim.12838 30264910

[43] McGuinness B Todd S Passmore P Bullock R. Blood pressure lowering in patients without prior cerebrovascular disease for prevention of cognitive impairment and dementia. Cochrane Database Syst Rev. (2009) 2009:CD004034. 10.1002/14651858.CD004034.pub3 19821318 PMC7163274

